# Supplementary material for: The Utility of Length of Mining Service and Latency in Predicting Silicosis among Claimants to a Compensation Trust
Source: Int J Environ Res Public Health. 2022 Mar 17;19(6):3562. doi: 10.3390/ijerph19063562 (PMC8953429; doi:10.3390/ijerph19063562)
Supplement: Supplementary file 1 [file ijerph-19-03562-s001.zip › ijerph-1614115-supplementary.pdf]

Supplementary material

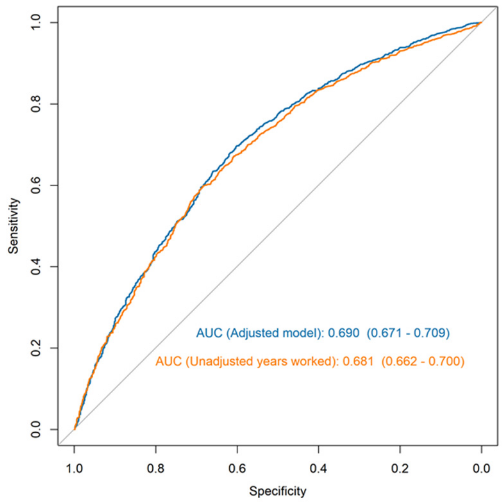

Figure S 1. Receiver operating characteristic curve for length of service (years) as predictor against compensable silicosis as outcome
